# The Anatomy of the Thymus Gland

**Published:** 1832-07-01

**Authors:** 


The Anatomy of the Thymus Gland. By *Sir Astley Cooper*, Bart. F.R.S. Serjeant Surgeon to the King, Consulting Surgeon to Guy's Hospital, &c. Quarto, bds. pp. 47; five lithograph Plates, price 14s. Longman's, London, 1832.

It requires no gift of prophecy to predict that Sir Astley Cooper's name will go down to posterity as the brightest, we say advisedly the very brightest, ornament of the science of surgery of the present century. We fear not the accusation of flattery in making this assertion, for such an accusation would be repelled by the body of the profession. The sin, the besetting sin, of the members of our profession is avarice, the sordid thirst of fees. For gold they sigh, and for gold they toil; ambition, "the last sin of noble minds," is with them but the lust of wealth, and place and pension are coveted but for it. When that is obtained, when age has destroyed the power, but not the wish, of obtaining more, when patients pass the door and crowd to the levee of another, the physician or surgeon, fashionable once but deservedly neglected now, sinks from the scene unhonored and unregarded, contemptible and contemned. The man of the world may smile at this, and may find in his pockets a refuge from contempt. Be it so. The consciousness of deserving the thanks of posterity, and the reception of the honest and hearty approbation of those of his own time, whose applause is sincere because it is unbought, gratifying because it is merited, and influential because it is equally bold and pure, must inspire in a generous mind such a train of feelings as would not be bartered for the gems of Peru. And let not the worldlings deceive themselves; a change is coming over our social and political relations, our public and our moral feelings, that will lessen most materially the influence of family and of faction, that will make even fees less accessible to the fingers of those who rely on such influence for their acquisition, that will give to industry, without interest, a fresh stimulus and a brighter prospect—to the press, that is to the symbol and the expression of public opinion, a higher power—to intrigue a narrower sphere—and to ingenuous emulation, and the passion for honest fame, a more brilliant guerdon.

These reflections have not inaptly been excited by the contemplation of the career of Sir Astley Cooper. Let would-be aristocratic doctors say what they will, he has proved himself an honour to the profession, nor do we feel shame in proclaiming it. To have been the most successful surgeon is little, for success might be owing to art or accident, or both. But to have written *the best book* on hernia—*the best book* on fractures and dislocations—*the best book* on the diseases of the breast—*the best book* on the diseases of the testis—and to have delivered *the best course* of surgical lectures, is no common honour, or series of honours. Sir Astley Cooper has devoted great attention to the anatomy of parts concerned in hernia, to the anatomy of the spleen, to the anatomy of the testis; and now we find him patiently, skilfully, and successfully investigating the anatomy of the thymus gland. Sir Astley Cooper has spent a youth of labour—is he spending an age of ease? We hesitate not to say that he is; we hesitate not to affirm that, to a mind like that of Sir Astley Cooper, useful, active, honourable professional employment is ease, satisfaction, delight. Such a mind would be corroded in a twelvemonth by the rust of sloth, would be gnawed Prometheus-like by the vulture of inaction; such a man would be miserable, nay he would not, could not exist, unsupported by the knowledge that he was working for the benefit of his species.

Sir Astley observes, that it is almost unnecessary to say that the subject is deserving of attention; "for every portion of the animal body, however minute, should be carefully traced and accurately known." Sir Astley commences with the description of the thymus gland in the fœtal calf, in which it appears that its form and dimensions are such as to render it very apt for examination. As our space, at this late period of the quarter, is too limited to allow of more than a brief review of the work, we must pass at once to the anatomy of the thymus in the human fœtus. We may state, however, for the benefit of those who are inclined to pursue the investigation, that the gland in the lamb and calf is more easily investigated, and made into preparations, than that of the dog, kitten, ass, pig, or any other animal examined by Sir Astley.

### Thymus Gland in the Human Subject.

"This Gland is formed of a thoracic and a cervical portion on each side. The former is situated in the anterior mediastinum, and the latter is placed in the neck just above the first bone of the sternum and behind the sterno hyoidei and sterno thyroidei muscles.

Between two and three months of fœtal life, as will be seen in the plate, it is so small as to be but just perceptible.

At three months its increase is in proportion to the relative magnitude of the fœtus, and thus it continues to grow gradually and equally to the seventh month, when it enlarges out of proportion to its former growth.

At eight months it is large, but at the ninth month has undergone a sudden change, becomes of great size, and is said to weigh half an ounce, from which circumstance, however, on account of the cavities which it contains and the varieties to which it is subject, no judgment of its bulk can be formed.

It increases after birth, and continues large to the first year, when it slowly disappears to the time of puberty; and in after age it ceases to have cavities, and becomes a body of very small dimensions." 19.

Sometimes a third thoracic lobe exists joining the other two, yet admitting of their separation under a careful dissection. Sir A. has seen the vena innominata pass through the gland, and he has seen it placed anteriorly to the cervical lobes. Its form is subject to considerable variation; the left gland is often larger than the right.

After giving a clear and accurate account of the relative situation of the gland, and of the fascia of the thorax, that fascia which may be said to form the roof of the thoracic cavity, as the diaphragm constitutes its floor, our author proceeds to the composition of the thymus.

It is formed of two distinct bodies, which may properly be called a right and left thymus gland. It is connected to the surrounding parts by an envelope of coarse cellular membrane, fixing it and uniting its two portions.

"When this membraneous covering is removed, the substance of the Gland is exposed, which is found to be of the conglomerate kind, being formed of numerous lobes which are connected together by a second covering of reticular tissue uniting the lobes to each other, and combining its parts by entering minutely into its interstices.

The lobes of this Gland differ in magnitude, but not one of them appears to be larger than a pea, and they vary from that of the head of a pin to the size above-mentioned. When the form of the Thymus is strictly investigated, the lobes are found to be disposed in a serpentine direction around a cavity hereafter to be described.

The Gland may be unravelled and it will be discovered to be composed of a rope on each side, of which the right and left Thymus is constituted, and on each of these the large lobes form knots, and it appears like a necklace of beads, but even these lobes may be still further separated.

In order to succeed in unravelling the Gland, it is necessary to divide the arteries, veins, as well as a mucous membrane, to be described hereafter, as the arteries, veins and membrane unite the lobes to each other to give them a serpentine course, to shorten the Gland, and to lessen the space which it would otherwise occupy.

These ropes are disposed in a spiral course around a central or nearly central cavity, and this disposition of them is preserved by the arteries, veins, and mucous membrane, by the division of which the ropes are unravelled.

The spiral rope which constitutes the Gland on the right side has no communication with that on the left, although the two Glands are combined into one by cellular tissue, yet in its usual formation, the glandular structure continues entirely separate.

In order to distinctly observe the rope, and to unravel it satisfactorily, it is necessary to dissect it, in part, in water, and then harden it in alcohol, when the dissection may be minutely pursued, and the lobes and their communicating portions be preserved and readily demonstrated.

This rope or chain of gland is composed of lobes of different sizes, connected together by membrane and by smaller portions of gland which surround a large internal cavity.

To proceed with the investigation of the structure of this Gland, remove a very thin superficial slice of each lobe, or of several of these, and numerous little cavities will be seen which may be set open after the organ has been hardened in spirits of wine, and these are the secretory cavities or cells producing the fluid which issues so abundantly.

The lobes being further examined, beside their cells, are found to contain a small pouch at their bases, which leads into a reservoir, so that the secretion which escapes from the lobes finds a ready entrance into the cavity of the Gland, from which it may be absorbed.

If a pipe be introduced into the Gland, and alcohol be injected, and the organ immersed in strong spirits, or a solution of alum, a large cavity will be filled, which I shall call the reservoir of the Thymus.

This reservoir forms a general communication between the different lobes; it begins from the inferior part of the thoracic portion, and extends from thence into the extremity of the cervical.

The reservoir does not maintain a straight course, but passes spirally, or in a serpentine direction, through the thoracic part of the Gland, and is somewhat more direct in the cervical portion.

With regard to its size, it varies in different places, but generally is the largest near the centre of the thoracic, and it is least at the communication of the cervical with the thoracic part of the Gland.

In the cervical portion it increases, but is less than in the thoracic, yet it still may be distinctly traced.

When opened, after having been injected and hardened, its internal surface appears to be lined by a smooth membrane; but if it be at once dissected in water, this lining membrane is found to be of the mucous kind, for it is rather villous than smooth, and instead of having a few red vessels, when filled with a vermillion injection, it is found to be highly vascular, and the arteries which are distributed to it may be seen meandering upon its surface and minutely dividing so as to redden every part of it.

Its interior forms ridges, which are produced by small ligamentous bands, which cross the surface of the reservoir in various directions and encircle the mouths of the pouches; these bands are formed for the purpose of keeping the lobes together, of preventing an injurious yielding of the parietes of the cavity, and to give strength to resist too great an accumulation of the secretion.

When the reservoir is floated in water, a number of small openings appear upon its internal surface, and if a probe be introduced into these, it passes into the pouch at the roots of the lobes, so that by these apertures, the secreted fluid escapes into the reservoir.

These orifices are not so numerous as the lobes themselves, the reason for which is, that each pouch communicates with more than one lobe.

The boundaries or walls of the Gland are full of secretory cavities or cells, which are extremely minute; they communicate with each other and open into the pouch of the lobes, and from the pouch into the reservoir." 30.

Sir Astley gives directions for injecting and preparing the gland, for which we must refer to the work itself.

With respect to the arteries. Each thoracic portion is supplied by a branch of the internal mammary, which passes to the inner side of the reservoir, and is distributed to the mucous membrane and glandular structure. The other principal artery of the thymus is sometimes derived from the superior thyroid, sometimes from the inferior; it is distributed to the cervical portion, and ultimately anastomoses with the branch from the internal mammary. These arteries besides supplying the gland with blood, serve to combine the lobes, for until they are divided the ropes cannot be unravelled. The principal *Venæ Thymicæ* end in the vena innominata, though small branches pass to the internal mammary and thyroidal veins. Sir A. has only been able to inject in the human subject the absorbent vessels proceeding from an absorbent gland of the thymus. Absorbent glands are found at the upper part of the sternum in the mediastinum; a small gland is also found between the thoracic portions, and some at the junction of the thymus with the jugular and subclavian veins, where the principal trunks of the absorbent vessels at all periods of life terminate. The advantages of comparative anatomy are here evident, for in the fœtal calf Sir Astley is able to fill with coarse injection two large absorbent trunks, passing nearly directly downwards on the posterior surface of the glands, converging on their approach towards the sternum, and terminating in the jugular veins at their junction with the superior cava by one or more orifices on each side.

The nerves of the thymus are very minute. They are probably derived from the superior thoracic ganglion of the sympathetic and the phrenic. Sir A. has seen a filament from the junction of the nervus vagus and sympathetic pass on the side of the thyroid gland to the thymus.

*Physiology of the Thymus Gland.* It is more than probable that this gland performs an office of some consequence in the fœtus. Hewson thought that it formed the internal part of the red globules of the blood, and that it was an appendage to the lymphatic glands, to which he considered it similar in structure and use. The former idea is pure fancy; the latter is demonstrably not the fact. Let us look at the secretion of the gland.

"It has been already stated, that this Gland secretes a great abundance of white fluid; that it is situated between the veins in which the great absorbent ducts of the body terminate; that to each cornu is attached a large absorbent duct in the fœtal calf, capable of being filled with coarse injection, and that this vessel terminates at the junction of the jugular veins in the vena innominata.

This fluid, although constantly found in the human fœtus, having the appearance of chyle, viz. white like cream, but with a small admixture of red globules, is not easily procured in sufficient quantity to make it the subject of chemical analysis; but from fœtal calves, two or three ounces may be without difficulty collected, and an abundant opportunity afforded of ascertaining its composition.

The best mode of obtaining it, is, by cutting the Gland into very small pieces, and placing them upon gauze, which being squeezed, the solid is separated from the fluid part, and the latter escapes through the gauze.

The Thymus should be previously immersed in water to deprive it of its blood.

The fluid thus collected from the calf, has the appearance of cream slightly tinged with blood, and to the eye, has the character of chyle.

*Warm water* dissolves a large portion of it.

*Heat* readily coagulates it.

*Alcohol* coagulates it.

*Sulphuric Acid* not only coagulates, but chars it.

*Nitric Acid* coagulates it firmly, first turning it white, and then yellow.

*Nitric Acid diluted*, precipitates a white solid from its solution in water, giving it the appearance of milk.

*Muriatic Acid* coagulates it firmly, and turns it white.

*Liquor Potassæ* converts it into a muco albuminous matter, which falls, in long extended threads, like the saliva in Ranula, and gives it much the appearance of white of egg." 39.

Collecting some of the secretion from the calf, Sir Astley sent it to Dr. Dowler of Richmond, already very favourably known by a paper in the Medico-Chirurgical Transactions (Vol. XII. Part I.) *On the Products of Acute Inflammation.* Sir Astley did not inform him of the nature of the fluid, but merely requested him to analyze it. The following was his reply.

" My Dear Sir Astley,—The fluid consists of the following substances, and which are placed in the order of their proportions.

100 parts contain 16 of solid matter. Incipient fibrin. Albumen.

Mucus and muco extractive matter. Salts consisting chiefly of muriate and phosphate of potash and phosphate of soda.

Of phosphoric acid a trace.

That the method of examination might be as unobjectionable as possible, the employment of active chemical reagents was avoided.

It was ascertained that the presence of the very minute quantity of free acid destroyed the affinity of the fibrinous particles for each other, for as soon as the acid was either saturated with an alkali, or further diluted with water, they then adhered together.

A portion of the fluid on being dropped into two or three times its weight of water, and gently stirred about will unite with a part of it, and after a short time be converted into a gelatinous looking mass. This mass consists of a solid and of a fluid part, and which may be separated from each other by mechanical means.

It is only necessary to inclose it in a fine linen rag, in such a manner that the latter may form a kind of loose bag around it, and then to gently rub portions of it between the finger and thumb, so as to break down the reticular tissue which retains the fluid portion. The bag must be occasionally gently pressed or carefully twisted, so as to separate this portion as it becomes liberated ; towards the end of the process more force may be used.

On opening the bag a viscid looking substance will be found, which when further pressed and dried, bears a close resemblance to ordinary fibrin, both in its chemical and physical properties. The linen bag used for the separation of the fibrin absorbs a quantity of the fluid part containing other animal matters. These must be separated by means of water, and added to the portion that had already passed through it ; when sufficiently concentrated under an exhausted receiver the albumen may be coagulated by heat, and in this way separated from the other animal and saline substances.

The other steps of the examination may be conducted in the usual manner.

I am sincerely yours, THOMAS DOWLER." 42.

If the fluid be examined in the microscope it is found to contain an immense number of white particles. The muco-extractive matter is probably derived from the mucous membrane lining the reservoir and secretory cavities of the gland.

" It appears to be an error to suppose that this Gland continues for some time after birth to perform the same office as that which it supported in the fœtal state.

I injected the Gland in a child of one month, and I found that the lobes had become quite thinned by absorption, although the reservoirs in part remained, but even one of these was partially obliterated. In a child of four months the reservoir was very small, broken into several portions, and the weight of the Gland which should have been in the fœtus of nine months 240 grains, weighed only 45 grains, or about five times less than in the fœtal state.

In a calf of four months, the Gland is very large, yet the cells and reservoirs will not receive one half of the injection which will enter in the fœtus of nine months.

I will therefore put the following query.

As the Thymus secretes all the parts of the blood, viz. albumen, fibrin and particles, is it not probable that the Gland is designed to prepare a fluid well fitted for the fœtal growth and nourishment from the blood of the mother before the birth of the fœtus, and consequently before chyle is formed from food, and this process continues for a short time after birth, the quantity of fluid secreted from the Thymus gradually declining as that of chylification becomes perfectly established ?" 44.

It appears to us that there is one objection to this idea. The secretion of "all the parts of the blood" is formed from the blood itself, and we have no satisfactory proof that the fluid secreted in the thymus, and transmitted into the venous system, is more fitted for nourishment than the arterialized blood from which it was produced. Arterial blood is the pabulum of life, and we know of no parallel instance in which a fluid, of more nutritious powers for the animal itself, is formed in and by that animal's organization. But we may be in error, and we leave Sir Astley's suggestion to the consideration of the profession.

*Disease of the Gland.*

" Parts which have ceased to perform their functions, as the mamma after menstruation and parturition, so frequently degenerate into diseased changes that morbid affections of this Gland might be expected to be frequent, yet in the course of more than forty years' experience, I have only witnessed one example of it.

Varieties in size are of common occurrence, but diseased changes of structure are extremely rare.

The following case occurred many years ago.

I was requested to visit a young person 19 years of age, who suffered under so severe a dyspnœa that it was with great difficulty she could remain recumbent for a few minutes, and if a short period of repose was obtained, she started up with a sense of suffocation, and for several seconds struggled violently for breath.

Upon enquiring into the cause of her suffering, I found a swelling which occupied the inferior part of the neck at the upper opening of the thorax, which projected above the clavicle upon each side, and as I supposed arose from an enlargement of the absorbent Glands at the termination of the jugular and subclavian veins.

The swelling had existed for many years, but of late suddenly increased. I ordered leeches to be applied, her bowels to be opened, and on the following day she was somewhat better, but another day brought with it not only her former, but still more aggravated sufferings ; I then advised a blister to the upper part of the sternum and to the swelling in the neck, desired the cuticle to be removed, the part to be dressed with the unguentum hydrargyri, and directed her to take calomel and opium, which she accomplished without much difficulty, as her deglutition was less affected than her breathing.

The means which I recommended gave her only slight temporary relief, and she became daily weaker, her legs were œdematous and she was unable to get any rest, but in the sitting posture, and then only with her head inclined forwards, and supported in that position by her sisters: for the moment it fell back, the pressure of the tumour on the trachea and the dyspnœa were suddenly increased.

I witnessed her making daily approaches to dissolution, without being able to afford her any permanent benefit: she died after a fortnight, not from any sudden attack of suffocation, but from being worn out by the constant irritation excited by the difficulty in respiration.

I obtained permission to examine the body, and found the disease was situated in the Thymus Gland ; the swelling reached from the curvature of the aorta to the lower part of the Thyroid Gland, and the latter was also considerably enlarged.

The Thymus appeared of a yellowish white colour, and was divided into several large lobes.

The trachea was involved in the tumour, and its sides were compressed by it, so that its transverse diameter was somewhat diminished. The arteria innominata was placed behind it, and the left subclavian, and left carotid arteries to its left side, it surrounded the vena innominata, and upon cutting into the vein, the diseased Gland was found projecting into its cavity, and upon making an incision into the swelling, the reticular texture of the Gland was found to be filled by a white pulpy substance.

In this case, the complaint was compounded of a diseased growth of the Thymus and of Bronchocele, or an unnatural growth of the Thyroid Gland. The latter is so placed that its enlargement little endangers suffocation, because the surrounding parts can yield to the pressure of the swollen Gland ; but as the Thymus is situated in the thoracic opening, in its enlarged state it soon reaches the sternum and first rib, by which it is bound, and therefore, its increase is towards the trachea, which becomes enveloped by it, and its function interrupted in consequence of its compression.

The disease appeared to be of the Fungoid kind." 47.

The preceding quotation terminates the work. After the observations which we made at the commencement of this article, we need say no more. We repeat our earnest wish, that the able and worthy author may long be spared to complete his career of useful investigation, and that the present will not be the last occasion, as it has not been the first, on which it becomes a duty to pay a well-merited tribute of applause to Sir Astley Cooper. The present work should be in the possession of all the medical book societies, as well as in the libraries of those who can afford to purchase standard productions.

